# Supplementary material for: MxDiffusion: A Physics-Aware Maxwell’s Law-Guided Diffusion Model Strategy for Inverse Photonic Metasurface Design
Source: Nano Lett. 2026 Apr 6;26(14):4897–905. doi: 10.1021/acs.nanolett.6c00943 (PMC13088363; doi:10.1021/acs.nanolett.6c00943)
Supplement: Supplementary file 1 [file nl6c00943_si_001.pdf]

Supporting Information for

MxDiffusion: A Physics-Aware Maxwell's Law-Guided Diffusion Model Strategy for Inverse Photonic Metasurface Design

Sujoy Mondal[1], Taehyuk Park[1], Sudipta Biswas[2], Alan X. Wang[2], Wenshan Cai[1,3]*

[1] School of Electrical and Computer Engineering, Georgia Institute of Technology, Atlanta, Georgia 30332, United States

[2] Department of Electrical and Computer Engineering, Baylor University, Waco, Texas 76798, United States

[3] School of Materials Science and Engineering, Georgia Institute of Technology, Atlanta, Georgia 30332, United States

*Correspondence to: wcai@gatech.edu

Contents:

1. Diffusion model overview
2. Detailed mathematics of the forward steps of the diffusion model
3. Detailed mathematics of the reverse steps of the diffusion model
4. Forward and reverse diffusion steps illustration
5. U-net model architecture
6. Gradual generation of electric fields from the gaussian noise
7. Gradual generation of final pattern from the gaussian noise
8. Timesteps selection and training procedure

1. Diffusion model overview

As discussed in the main text, a diffusion model operates through two main phases: forward diffusion and reverse diffusion. At the initial forward stage, the clean image is denoted by x_0. At the first timestep ($t = 1$), Gaussian noise $\varepsilon_1 \sim \mathcal{N}(0,I)$ is added to the image according to

$$x_1 = \sqrt{\alpha_1}x_0 + \sqrt{1-\alpha_1}\varepsilon_1 = \sqrt{\alpha_1}x_0 + \sqrt{\beta_1}\varepsilon_1 \tag{S1}$$

where the definitions of α_1 and β_1 are provided below. Similarly, at an arbitrary timestep t, the noisy image x_t is obtained from the previous image x_{t-1} by

$$x_t = \sqrt{\alpha_t}x_{t-1} + \sqrt{1-\alpha_t}\varepsilon_t = \sqrt{\alpha_t}x_{t-1} + \sqrt{\beta_t}\varepsilon_t \tag{S2}$$

Here, $\{\beta_1,\beta_2,\ldots,\beta_T\}$ is a sequence of small positive values satisfying $0 < \beta_t \ll 1$, and $\alpha_t = 1 - \beta_t$. The parameter β_t controls the amount of new noise added at timestep t, while α_t determines how much of the original signal is retained at that step. It can be shown mathematically (see Section 2) that the noisy image at timestep t can be directly expressed in terms of the original clean image x_0 as

$$x_t = \sqrt{\overline{\alpha}_t}x_0 + \sqrt{1-\overline{\alpha}_t}\varepsilon \tag{S3}$$

where $\overline{\alpha}_t = \prod_{s=1}^{t} \alpha_s$

In this expression, $\sqrt{\overline{\alpha}_t}x_0$ represents the remaining clean signal, while $\sqrt{1-\overline{\alpha}_t}\varepsilon$ corresponds to the accumulated noise after t steps. Typically, the total number of timesteps T is chosen in the range of 500–1000, ensuring that only a small amount of noise is added at each step. By the final timestep ($t = T$), the image is transformed into nearly pure Gaussian noise. Since the forward diffusion process is entirely defined by fixed equations with predefined α_t and β_t, it does not involve any learnable parameters or neural network training.

As discussed in the main text, the reverse diffusion process is responsible for generating new images by progressively removing noise from a noisy image, as illustrated in Figure S1. This process starts with a highly noisy image and reconstructs a clean sample through a sequence of denoising steps. The reverse update at timestep t is given by

$$x_{t-1} = \frac{1}{\sqrt{\alpha_t}}\left(x_t - \frac{\beta_t}{\sqrt{1-\bar{\alpha}_t}}\varepsilon_\theta(x_t,t)\right) + \sigma_t z \tag{S4}$$

This equation describes how to obtain a less noisy image x_{t-1} from the current noisy image x_t. Here, $\varepsilon_\theta(x_t,t)$ is the noise predicted by the neural network model. While noise is added explicitly during the forward diffusion process using Eq. S3, the reverse process relies on a U-Net–based neural network, as shown in Figure S2, to predict this noise. $\sigma_t z$, the stochasticity term, where $z \sim \mathcal{N}(0,I)$, introduces controlled randomness into the reverse process. This term enables proper sampling from the learned distribution and prevents the model from collapsing to a single deterministic reconstruction.

As shown in Figure S2, during training, the U-Net neural network learns to predict the noise ε_θ given the noisy image x_t, the diffusion timestep t, and additional conditional inputs such as the transmission spectrum or the electric field corresponding to the original image x_0. Reverse sampling is performed over T timesteps. The process begins with a randomly sampled noise image x_T. At timestep T, the trained model predicts the noise using the conditional input and timestep information, and Eq. S4 is applied to obtain x_{T-1}. This procedure is repeated sequentially for timesteps $T-1, T-2, \ldots, 1$ each time using the same conditional input, until the final clean image x_0 is obtained. The conditional input therefore guides the reverse diffusion process, ensuring that the generated image satisfies the desired physical or spectral constraints. Other generative models typically attempt to generate images from target inputs in a single step, whereas diffusion models

perform generation through T sequential timesteps. By decomposing image generation into a series of small denoising tasks, diffusion models transform a complex generation problem into multiple simpler noise-prediction problems, leading to more stable training and improved sample quality.

2. Detailed mathematics of the forward steps of the diffusion model

We described the working principle of diffusion model in section 1. In this section we will go through the mathematical part in more detail. As shown before, the forward diffusion process progressively corrupts a clean sample x_0 by adding Gaussian noise over T discrete timesteps. We will expand the original mathematics from ref [1].

The one-step transition is defined as:

$$x_t = \sqrt{\alpha_t}\, x_{t-1} + \sqrt{\beta_t}\, \epsilon_t,\ \epsilon_t \sim \mathcal{N}(0,I),$$

Where,

$\alpha_t = 1 - \beta_t$,$0 < \beta_t \ll 1$.We will prove this equation here.

<u>Step 1: Unrolling a Few Steps</u>

For $t = 1$:

$$x_1 = \sqrt{\alpha_1}\, x_0 + \sqrt{\beta_1}\, \epsilon_1$$

For $t = 2$:

$$x_2 = \sqrt{\alpha_2}\, x_1 + \sqrt{\beta_2}\, \epsilon_2$$

Now we substitute x_1:

$$x_2 = \sqrt{\alpha_2}\left(\sqrt{\alpha_1}\,x_0 + \sqrt{\beta_1}\,\epsilon_1\right) + \sqrt{\beta_2}\,\epsilon_2$$
$$= \sqrt{\alpha_2\alpha_1}\,x_0 + \sqrt{\alpha_2\beta_1}\,\epsilon_1 + \sqrt{\beta_2}\,\epsilon_2$$

For $t = 3$:

$$x_3 = \sqrt{\alpha_3}\,x_2 + \sqrt{\beta_3}\,\epsilon_3$$

Now we substitute x_2:

$$x_3 = \sqrt{\alpha_3\alpha_2\alpha_1}\,x_0 + \sqrt{\alpha_3\alpha_2\beta_1}\,\epsilon_1 + \sqrt{\alpha_3\beta_2}\,\epsilon_2 + \sqrt{\beta_3}\,\epsilon_3$$

From this expansion, we observe the general pattern:

$$x_t = \sqrt{\left(\prod_{s=1}^{t} \alpha_s\right)}\,x_0 + \sum_{k=1}^{t}\left(\sqrt{\beta_k}\prod_{s=k+1}^{t}\sqrt{\alpha_s}\right)\epsilon_k$$

Now we can define the cumulative product:

$$\grave{\alpha}_t = \prod_{s=1}^{t} \alpha_s$$

So, the signal term becomes:

$$\sqrt{\grave{\alpha}_t}\,x_0$$

Step 2: Showing the Entire Noise Sum is One Gaussian

We can define the total noise term:

$$\eta_t = \sum_{k=1}^{t} c_{t,k}\, \epsilon_k$$

Where,

$$c_{t,k} = \sqrt{\beta_k} \prod_{s=k+1}^{t} \sqrt{\alpha_s}$$

Each $\epsilon_k \sim \mathcal{N}(0,I)$ and they are independent.

A linear combination of independent Gaussians is Gaussian:

$$\eta_t \sim \mathcal{N}\left(0, \left(\sum_{k=1}^{t} c_{t,k}^2\right) I\right)$$

Now we can compute the variance

$$\sum_{k=1}^{t} c_{t,k}^2 = \sum_{k=1}^{t} \beta_k \prod_{s=k+1}^{t} \alpha_s$$

A key identity (provable by induction, see below):

$$\sum_{k=1}^{t} \beta_k \prod_{s=k+1}^{t} \alpha_s = 1 - \grave{\alpha}_t$$

The proof by induction is shown later in this section.

Therefore,

$$\eta_t \sim \mathcal{N}\big(0,(1-\grave{\alpha}_t)I\big)$$

Hence, we can write:

$$\eta_t = \sqrt{1-\grave{\alpha}_t}\,\epsilon, \epsilon \sim \mathcal{N}(0,I)$$

Final Result

Substituting back into the expression for x_t:

$$\boxed{x_t = \sqrt{\grave{\alpha}_t}\,x_0 + \sqrt{1-\grave{\alpha}_t}\,\epsilon, \epsilon \sim \mathcal{N}(0,I)}$$

Interpretation

- $\sqrt{\grave{\alpha}_t} x_0$ is the remaining clean signal.
- $\sqrt{1-\grave{\alpha}_t}\epsilon$ is the accumulated Gaussian noise.
- When $\grave{\alpha}_T \approx 0$, we get:

$$x_T \sim \mathcal{N}(0,I)$$

meaning the sample becomes nearly pure Gaussian noise.

Proof by Induction

We want to prove, for $t \geq 1$,

$$\sum_{k=1}^{t} \beta_k \prod_{s=k+1}^{t} \alpha_s \;=\; 1-\grave{\alpha}_t, \text{where} \grave{\alpha}_t = \prod_{s=1}^{t} \alpha_s\,, \;\; \alpha_t = 1-\beta_t.$$

Base case: $t = 1$

Left-hand side:

$$\sum_{k=1}^{1} \beta_k \prod_{s=k+1}^{1} \alpha_s = \beta_1 \prod_{s=2}^{1} \alpha_s .$$

The product over an empty index set equals 1, so this is β_1.

Right-hand side:

$$1 - \grave{\alpha}_1 = 1 - \alpha_1 = 1 - (1 - \beta_1) = \beta_1.$$

So, the identity holds for $t = 1$.

Inductive hypothesis

Now we assume the identity holds for some $t \geq 1$:

$$\sum_{k=1}^{t} \beta_k \prod_{s=k+1}^{t} \alpha_s \;=\; 1 - \grave{\alpha}_t.$$

Inductive step: prove it for $t + 1$

We consider the left-hand side at $t + 1$:

$$\sum_{k=1}^{t+1} \beta_k \prod_{s=k+1}^{t+1} \alpha_s = \left(\sum_{k=1}^{t} \beta_k \prod_{s=k+1}^{t+1} \alpha_s \right) + \beta_{t+1} \prod_{s=t+2}^{t+1} \alpha_s .$$

Again, the last product is empty, so it equals 1. Thus:

$$= \sum_{k=1}^{t} \beta_k \left(\prod_{s=k+1}^{t} \alpha_s \right) \alpha_{t+1} + \beta_{t+1}.$$

Factoring out α_{t+1} from the sum:

$$= \alpha_{t+1} \sum_{k=1}^{t} \beta_k \prod_{s=k+1}^{t} \alpha_s + \beta_{t+1}.$$

Now applying the inductive hypothesis:

$$= \alpha_{t+1}(1 - \grave{\alpha}_t) + \beta_{t+1}.$$

Now we expand and use $\beta_{t+1} = 1 - \alpha_{t+1}$:

$$= \alpha_{t+1} - \alpha_{t+1}\grave{\alpha}_t + (1 - \alpha_{t+1}) = 1 - \alpha_{t+1}\grave{\alpha}_t.$$

Finally, we note

$$\grave{\alpha}_{t+1} = \prod_{s=1}^{t+1} \alpha_s = \left(\prod_{s=1}^{t} \alpha_s \right) \alpha_{t+1} = \grave{\alpha}_t \, \alpha_{t+1},$$

So,

$$1 - \alpha_{t+1}\grave{\alpha}_t = 1 - \grave{\alpha}_{t+1}.$$

Thus, the identity holds for $t + 1$. By induction, it holds for all $t \geq 1$.

3. Detailed mathematics of the reverse steps of the diffusion model

The reverse process generates a sample by starting from Gaussian noise $x_T \sim \mathcal{N}(0,I)$ and iteratively sampling.

$$p_\theta(x_{t-1}|x_t) = \mathcal{N}\big(x_{t-1}; \mu_\theta(x_t,t,\text{cond}), \Sigma_\theta(t)\big), t = T,...,1.$$

Here, cond denotes conditioning information (e.g., target spectrum and/or electric field).

We can derive that reverse-sampling update equation in three clean steps:

Step 1: Starting from the true posterior $q(x_{t-1}|x_t,x_0)$ (Gaussian)

Forward process:

$$q(x_t|x_{t-1}) = \mathcal{N}\big(\sqrt{\alpha_t}\,x_{t-1}, \beta_t I\big), \alpha_t = 1 - \beta_t$$

and marginal:

$$q(x_t|x_0) = \mathcal{N}\big(\sqrt{\grave{\alpha}_t}\,x_0, (1-\grave{\alpha}_t)I\big), \grave{\alpha}_t = \prod_{s=1}^{t} \alpha_s$$

A standard DDPM result (from multiplying Gaussians) is:

$$q(x_{t-1}|x_t,x_0) = \mathcal{N}\big(\tilde{\mu}(x_t,x_0), \tilde{\beta}_t I\big)$$

With,

$$\tilde{\beta}_t = \frac{1-\grave{\alpha}_{t-1}}{1-\grave{\alpha}_t}\,\beta_t$$

$$\tilde{\mu}(x_t,x_0) = \frac{\sqrt{\grave{\alpha}_{t-1}}\,\beta_t}{1-\grave{\alpha}_t}\,x_0 + \frac{\sqrt{\alpha_t}(1-\grave{\alpha}_{t-1})}{1-\grave{\alpha}_t}\,x_t$$

So, the ideal reverse sampling step would be:

$$x_{t-1} = \tilde{\mu}(x_t, x_0) + \sigma_t z, z \sim \mathcal{N}(0, I)$$

(often $\sigma_t^2 = \tilde{\beta}_t$).

Step 2: Expressing x_0 using the noise ϵ

From the forward closed form:

$$x_t = \sqrt{\grave{\alpha}_t}\, x_0 + \sqrt{1 - \grave{\alpha}_t}\, \epsilon, \epsilon \sim \mathcal{N}(0, I)$$

solve for x_0:

$$x_0 = \frac{x_t - \sqrt{1 - \grave{\alpha}_t}\, \epsilon}{\sqrt{\grave{\alpha}_t}}$$

In practice ϵ is unknown, so the model predicts it:

$$\epsilon \approx \epsilon_\theta(x_t, t, \text{cond})$$

giving

$$\hat{x}_0 = \frac{x_t - \sqrt{1 - \grave{\alpha}_t}\, \epsilon_\theta(x_t, t, \text{cond})}{\sqrt{\grave{\alpha}_t}}$$

Step 3: Plug $\hat{x}_0$ into the posterior mean and simplify

Start with $\tilde{\mu}(x_t, x_0)$, substitute $x_0 = \hat{x}_0$:

$$\mu_\theta(x_t, t) = \frac{\sqrt{\grave{\alpha}_{t-1}}\, \beta_t}{1 - \grave{\alpha}_t}\, \hat{x}_0 + \frac{\sqrt{\alpha_t}(1 - \grave{\alpha}_{t-1})}{1 - \grave{\alpha}_t}\, x_t$$

Now inserting

$$\hat{x}_0 = \frac{x_t - \sqrt{1 - \grave{\alpha}_t}\epsilon_\theta}{\sqrt{\grave{\alpha}_t}}$$

and using

$$\grave{\alpha}_t = \alpha_t \grave{\alpha}_{t-1} \Rightarrow \sqrt{\grave{\alpha}_{t-1}}/\sqrt{\grave{\alpha}_t} = 1/\sqrt{\alpha_t}:$$

$$\mu_\theta(x_t,t) = \frac{\beta_t}{(1 - \grave{\alpha}_t)\sqrt{\alpha_t}}\left(x_t - \sqrt{1 - \grave{\alpha}_t}\,\epsilon_\theta\right) + \frac{\sqrt{\alpha_t}(1 - \grave{\alpha}_{t-1})}{1 - \grave{\alpha}_t}\, x_t$$

Now we group the x_t terms. The coefficient on x_t becomes:

$$\frac{\beta_t}{(1 - \grave{\alpha}_t)\sqrt{\alpha_t}} + \frac{\sqrt{\alpha_t}(1 - \grave{\alpha}_{t-1})}{1 - \grave{\alpha}_t} = \frac{\beta_t + \alpha_t(1 - \grave{\alpha}_{t-1})}{(1 - \grave{\alpha}_t)\sqrt{\alpha_t}}$$

But since $\grave{\alpha}_t = \alpha_t \grave{\alpha}_{t-1}$,

$$\beta_t + \alpha_t(1 - \grave{\alpha}_{t-1}) = (1 - \alpha_t) + \alpha_t - \alpha_t \grave{\alpha}_{t-1} = 1 - \grave{\alpha}_t$$

So, the coefficient simplifies to $\frac{1}{\sqrt{\alpha_t}}$.

The noise term becomes:

$$-\frac{\beta_t}{(1 - \grave{\alpha}_t)\sqrt{\alpha_t}}\sqrt{1 - \grave{\alpha}_t}\,\epsilon_\theta = -\frac{\beta_t}{\sqrt{\alpha_t}\sqrt{1 - \grave{\alpha}_t}}\,\epsilon_\theta$$

Therefore,

$$\boxed{\mu_\theta(x_t,t) = \frac{1}{\sqrt{\alpha_t}}\left(x_t - \frac{\beta_t}{\sqrt{1 - \grave{\alpha}_t}}\,\epsilon_\theta(x_t,t,\text{cond})\right)}$$

Finally, adding Gaussian noise in sampling:

$x_{t-1} = \frac{1}{\sqrt{\alpha_t}} \left(x_t - \frac{\beta_t}{\sqrt{1 - \grave{\alpha}_t}} \epsilon_\theta(x_t, t, \text{cond}) \right) + \sigma_t z, z \sim \mathcal{N}(0, I)$ This is the reverse sampling equation used in the main text.

4. Forward and reverse diffusion steps illustration

Figure S1 illustrates the diffusion model forward and reverse steps. The figure shows how noise is gradually added to the sample to make it a pure gaussian noise. Also, it illustrates how new sample can be generated from pure gaussian noise in the reverse steps.

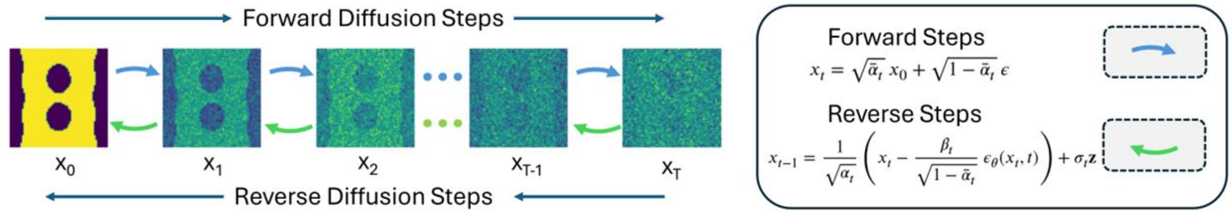

Figure S1: Illustration of the diffusion process, showing progressive noise addition during the forward steps (left to right) and iterative denoising during the reverse steps (right to left), along with the corresponding governing equations.

5. U-net model architecture

The diffusion model uses U-net model architecture to predict the noise at each steps of the reverse sampling. The main model of the framework is a conditional diffusion U-Net designed to predict the noise term in a DDPM process for a 33×33 single-channel electric field conditioned on a 120-point transmission spectrum. The model takes three inputs: a noisy field x_t, a timestep t,

and a conditioning vector y. Its output is a prediction of the noise, ϵ, added at timestep t, which is then used in the reverse diffusion process.

The conditioning mechanism is built around two embeddings: one for the diffusion timestep and one for the spectrum. The timestep embedding uses sinusoidal positional encoding. The scalar timestep is first normalized to the range [0, 1], then mapped into a high-dimensional representation using sine and cosine frequency functions. This produces a smooth, continuous representation of diffusion time. That embedding is then refined through a small MLP to produce a 128-dimensional time embedding.

The spectral conditioning vector, which has dimension 120, is passed through its own MLP. This network projects the spectrum into the same 128-dimensional latent space as the time embedding. The model then concatenates the time embedding and the spectrum embedding and projects the combined vector back to 128 dimensions using another linear layer. This fused embedding acts as the global conditioning vector that modulates the entire U-Net.

Conditioning is injected into the network through FiLM-based residual blocks. In each residual block, the fused conditioning embedding is transformed into scaling and shifting parameters (gamma and beta). These parameters are applied after group normalization inside the block, effectively allowing the timestep and spectrum to dynamically control the behavior of convolutional features at every resolution level. This is a powerful and flexible conditioning mechanism because it modulates intermediate activations rather than only influencing the input or output layers.

The spatial input first passes through a small preprocessing stage. Because the U-Net uses multiple downsampling layers, the input is padded so that its height and width become multiples of eight. A 33×33 field is therefore padded to 40×40. If coordinate channels are enabled, two

additional channels representing normalized x and y spatial coordinates are concatenated to the input. This gives the network explicit spatial awareness, which is particularly useful in physics-based field prediction tasks where boundary conditions matter.

The backbone of the architecture is a U-Net with four resolution levels. Starting from a base width of 96 channels, the number of feature channels doubles at each downsampling stage, reaching up to 768 channels in the bottleneck. The encoder path consists of FiLM-conditioned residual blocks followed by strided convolutions for spatial downsampling. At intermediate resolution (after the second downsampling), a multi-head self-attention block is inserted. This attention mechanism allows the model to capture long-range spatial dependencies, such as symmetry or global field structure, which standard convolutions might struggle to model.

At the lowest resolution (the bottleneck), two additional FiLM residual blocks further process the representation. The decoder path mirrors the encoder. Each stage begins with a transposed convolution to upsample the feature map, then concatenates the corresponding skip connection from the encoder. A FiLM residual block fuses these features, maintaining both high-level context and fine spatial details. This symmetric structure preserves spatial information while allowing deep, global reasoning.

The final output head applies group normalization and a 1×1 convolution to reduce the feature dimension back to a single channel. After cropping away the earlier padding, the output has shape (B, 1, 33, 33), where B is the batch size. Importantly, this output represents predicted noise, not the denoised image itself.

The model also incorporates classifier-free guidance (CFG). During training, the conditioning vector can be randomly replaced with a learned null embedding. This trains the network to operate both conditionally and unconditionally. At inference time, the model can compute both conditional

and unconditional predictions and combine them to strengthen conditioning through a guidance scale factor. This mechanism increases the influence of the spectrum during generation without changing the model architecture.

Overall, this architecture combines convolutional inductive bias, multi-resolution feature extraction, global attention, FiLM-based conditioning, coordinate awareness, and classifier-free guidance. It is specifically tailored for conditional generation of structured physical fields from spectral inputs, balancing expressive capacity with controlled parameter growth. The detailed model architecture is listed below. The model is named ImprovedUnet33.

```
ImprovedUnet33(
  (time_mlp): Sequential(
    (0): SinusoidalPositionEmbeddings()
    (1): Linear(in_features=128, out_features=128, bias=True)
    (2): SiLU()
  )
  (label_mlp): Sequential(
    (0): Linear(in_features=120, out_features=128, bias=True)
    (1): SiLU()
    (2): Linear(in_features=128, out_features=128, bias=True)
  )
  (emb_proj): Sequential(
    (0): SiLU()
    (1): Linear(in_features=256, out_features=128, bias=True)
  )
  (stem): Conv2d(3, 96, kernel_size=(3, 3), stride=(1, 1), padding=(1, 1))
  (down1): FiLMResBlock(
    (norm1): GroupNorm(8, 96, eps=1e-05, affine=True)
    (conv1): Conv2d(96, 96, kernel_size=(3, 3), stride=(1, 1), padding=(1, 1))
    (norm2): GroupNorm(8, 96, eps=1e-05, affine=True)
    (dropout): Dropout2d(p=0.1, inplace=False)
    (conv2): Conv2d(96, 96, kernel_size=(3, 3), stride=(1, 1), padding=(1, 1))
    (film): Sequential(
      (0): SiLU()
      (1): Linear(in_features=128, out_features=192, bias=True)
    )
```

```
        (skip): Identity()
        (act): SiLU()
      )
      (ds1): Downsample(
        (op): Conv2d(96, 96, kernel_size=(4, 4), stride=(2, 2), padding=(1, 1))
      )
      (down2): FiLMResBlock(
        (norm1): GroupNorm(8, 96, eps=1e-05, affine=True)
        (conv1): Conv2d(96, 192, kernel_size=(3, 3), stride=(1, 1), padding=(1, 1))
        (norm2): GroupNorm(8, 192, eps=1e-05, affine=True)
        (dropout): Dropout2d(p=0.1, inplace=False)
        (conv2): Conv2d(192, 192, kernel_size=(3, 3), stride=(1, 1), padding=(1, 1))
        (film): Sequential(
          (0): SiLU()
          (1): Linear(in_features=128, out_features=384, bias=True)
        )
        (skip): Conv2d(96, 192, kernel_size=(1, 1), stride=(1, 1))
        (act): SiLU()
      )
      (ds2): Downsample(
        (op): Conv2d(192, 192, kernel_size=(4, 4), stride=(2, 2), padding=(1, 1))
      )
      (attn_mid): SelfAttention2d(
        (norm): GroupNorm(8, 192, eps=1e-05, affine=True)
        (attn): MultiheadAttention(
          (out_proj):                 NonDynamicallyQuantizableLinear(in_features=192,
out_features=192, bias=True)
        )
      )
      (down3): FiLMResBlock(
        (norm1): GroupNorm(8, 192, eps=1e-05, affine=True)
        (conv1): Conv2d(192, 384, kernel_size=(3, 3), stride=(1, 1), padding=(1, 1))
        (norm2): GroupNorm(8, 384, eps=1e-05, affine=True)
        (dropout): Dropout2d(p=0.1, inplace=False)
        (conv2): Conv2d(384, 384, kernel_size=(3, 3), stride=(1, 1), padding=(1, 1))
        (film): Sequential(
          (0): SiLU()
          (1): Linear(in_features=128, out_features=768, bias=True)
        )
        (skip): Conv2d(192, 384, kernel_size=(1, 1), stride=(1, 1))
```

```
    (act): SiLU()
  )
  (ds3): Downsample(
    (op): Conv2d(384, 384, kernel_size=(4, 4), stride=(2, 2), padding=(1, 1))
  )
  (down4): FiLMResBlock(
    (norm1): GroupNorm(8, 384, eps=1e-05, affine=True)
    (conv1): Conv2d(384, 768, kernel_size=(3, 3), stride=(1, 1), padding=(1, 1))
    (norm2): GroupNorm(8, 768, eps=1e-05, affine=True)
    (dropout): Dropout2d(p=0.1, inplace=False)
    (conv2): Conv2d(768, 768, kernel_size=(3, 3), stride=(1, 1), padding=(1, 1))
    (film): Sequential(
      (0): SiLU()
      (1): Linear(in_features=128, out_features=1536, bias=True)
    )
    (skip): Conv2d(384, 768, kernel_size=(1, 1), stride=(1, 1))
    (act): SiLU()
  )
  (mid1): FiLMResBlock(
    (norm1): GroupNorm(8, 768, eps=1e-05, affine=True)
    (conv1): Conv2d(768, 768, kernel_size=(3, 3), stride=(1, 1), padding=(1, 1))
    (norm2): GroupNorm(8, 768, eps=1e-05, affine=True)
    (dropout): Dropout2d(p=0.1, inplace=False)
    (conv2): Conv2d(768, 768, kernel_size=(3, 3), stride=(1, 1), padding=(1, 1))
    (film): Sequential(
      (0): SiLU()
      (1): Linear(in_features=128, out_features=1536, bias=True)
    )
    (skip): Identity()
    (act): SiLU()
  )
  (mid2): FiLMResBlock(
    (norm1): GroupNorm(8, 768, eps=1e-05, affine=True)
    (conv1): Conv2d(768, 768, kernel_size=(3, 3), stride=(1, 1), padding=(1, 1))
    (norm2): GroupNorm(8, 768, eps=1e-05, affine=True)
    (dropout): Dropout2d(p=0.1, inplace=False)
    (conv2): Conv2d(768, 768, kernel_size=(3, 3), stride=(1, 1), padding=(1, 1))
    (film): Sequential(
      (0): SiLU()
      (1): Linear(in_features=128, out_features=1536, bias=True)
```

```
        )
        (skip): Identity()
        (act): SiLU()
      )
      (us3): Upsample(
        (op): ConvTranspose2d(768, 768, kernel_size=(4, 4), stride=(2, 2), padding=(1,
1))
      )
      (up3): FiLMResBlock(
        (norm1): GroupNorm(8, 1152, eps=1e-05, affine=True)
        (conv1): Conv2d(1152, 384, kernel_size=(3, 3), stride=(1, 1), padding=(1, 1))
        (norm2): GroupNorm(8, 384, eps=1e-05, affine=True)
        (dropout): Dropout2d(p=0.1, inplace=False)
        (conv2): Conv2d(384, 384, kernel_size=(3, 3), stride=(1, 1), padding=(1, 1))
        (film): Sequential(
          (0): SiLU()
          (1): Linear(in_features=128, out_features=768, bias=True)
        )
        (skip): Conv2d(1152, 384, kernel_size=(1, 1), stride=(1, 1))
        (act): SiLU()
      )
      (us2): Upsample(
        (op): ConvTranspose2d(384, 384, kernel_size=(4, 4), stride=(2, 2), padding=(1,
1))
      )
      (up2): FiLMResBlock(
        (norm1): GroupNorm(8, 576, eps=1e-05, affine=True)
        (conv1): Conv2d(576, 192, kernel_size=(3, 3), stride=(1, 1), padding=(1, 1))
        (norm2): GroupNorm(8, 192, eps=1e-05, affine=True)
        (dropout): Dropout2d(p=0.1, inplace=False)
        (conv2): Conv2d(192, 192, kernel_size=(3, 3), stride=(1, 1), padding=(1, 1))
        (film): Sequential(
          (0): SiLU()
          (1): Linear(in_features=128, out_features=384, bias=True)
        )
        (skip): Conv2d(576, 192, kernel_size=(1, 1), stride=(1, 1))
        (act): SiLU()
      )
      (us1): Upsample(
```

```
        (op): ConvTranspose2d(192, 192, kernel_size=(4, 4), stride=(2, 2), padding=(1,
1))
      )
      (up1): FiLMResBlock(
        (norm1): GroupNorm(8, 288, eps=1e-05, affine=True)
        (conv1): Conv2d(288, 96, kernel_size=(3, 3), stride=(1, 1), padding=(1, 1))
        (norm2): GroupNorm(8, 96, eps=1e-05, affine=True)
        (dropout): Dropout2d(p=0.1, inplace=False)
        (conv2): Conv2d(96, 96, kernel_size=(3, 3), stride=(1, 1), padding=(1, 1))
        (film): Sequential(
          (0): SiLU()
          (1): Linear(in_features=128, out_features=192, bias=True)
        )
        (skip): Conv2d(288, 96, kernel_size=(1, 1), stride=(1, 1))
        (act): SiLU()
      )
      (out_norm): GroupNorm(8, 96, eps=1e-05, affine=True)
      (out_conv): Conv2d(96, 1, kernel_size=(1, 1), stride=(1, 1))
    )
```

The model architecture can be roughly presented as figure S2.

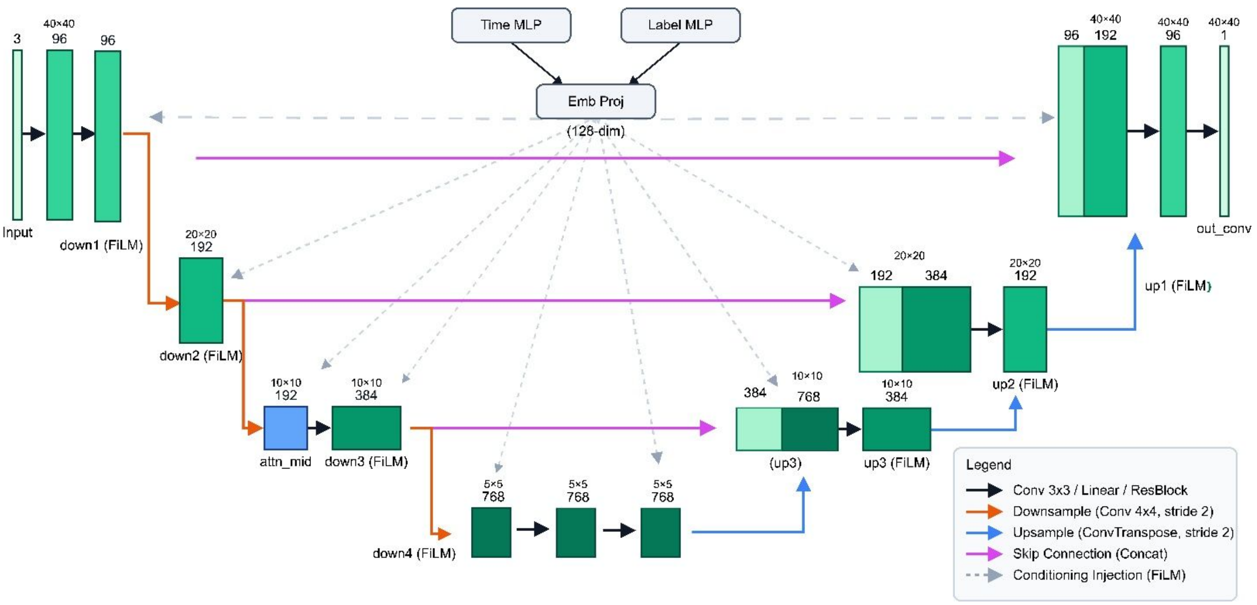

Figure S2: Architecture of the U-Net–based diffusion model, where the inputs include the noisy image at timestep $t(x_t)$, the timestep value, and conditional information such as transmission spectra; the network predicts the noise at this stage, which is then used to obtain the less noisy sample (x_{t-1}). The architecture shows how the layer shapes changes as the model undergoes downsampling followed by upsampling.

The model summary is as follows:

```
===========================================================================================
Layer (type:depth-idx)                        Output Shape              Param #
===========================================================================================
ImprovedUnet33                                [1, 1, 33, 33]            120
├─Sequential: 1-1                             [1, 128]                  --
│    └─SinusoidalPositionEmbeddings: 2-1      [1, 128]                  --
│    └─Linear: 2-2                            [1, 128]                  16,512
│    └─SiLU: 2-3                              [1, 128]                  --
├─Sequential: 1-2                             [1, 128]                  --
│    └─Linear: 2-4                            [1, 128]                  15,488
│    └─SiLU: 2-5                              [1, 128]                  --
│    └─Linear: 2-6                            [1, 128]                  16,512
├─Sequential: 1-3                             [1, 128]                  --
│    └─SiLU: 2-7                              [1, 256]                  --
│    └─Linear: 2-8                            [1, 128]                  32,896
├─Conv2d: 1-4                                 [1, 96, 40, 40]           2,688
├─FiLMResBlock: 1-5                           [1, 96, 40, 40]           --
│    └─GroupNorm: 2-9                         [1, 96, 40, 40]           192
│    └─SiLU: 2-10                             [1, 96, 40, 40]           --
│    └─Conv2d: 2-11                           [1, 96, 40, 40]           83,040
│    └─Sequential: 2-12                       [1, 192]                  --
│    │    └─SiLU: 3-1                         [1, 128]                  --
│    │    └─Linear: 3-2                       [1, 192]                  24,768
│    └─GroupNorm: 2-13                        [1, 96, 40, 40]           192
│    └─SiLU: 2-14                             [1, 96, 40, 40]           --
```

```
│    └─Dropout2d: 2-15                             [1, 96, 40, 40]          --
│    └─Conv2d: 2-16                                [1, 96, 40, 40]          83,040
│    └─Identity: 2-17                              [1, 96, 40, 40]          --
├─Downsample: 1-6                                  [1, 96, 20, 20]          --
│    └─Conv2d: 2-18                                [1, 96, 20, 20]          147,552
├─FiLMResBlock: 1-7                                [1, 192, 20, 20]         --
│    └─GroupNorm: 2-19                             [1, 96, 20, 20]          192
│    └─SiLU: 2-20                                  [1, 96, 20, 20]          --
│    └─Conv2d: 2-21                                [1, 192, 20, 20]         166,080
│    └─Sequential: 2-22                            [1, 384]                 --
│    │    └─SiLU: 3-3                              [1, 128]                 --
│    │    └─Linear: 3-4                            [1, 384]                 49,536
│    └─GroupNorm: 2-23                             [1, 192, 20, 20]         384
│    └─SiLU: 2-24                                  [1, 192, 20, 20]         --
│    └─Dropout2d: 2-25                             [1, 192, 20, 20]         --
│    └─Conv2d: 2-26                                [1, 192, 20, 20]         331,968
│    └─Conv2d: 2-27                                [1, 192, 20, 20]         18,624
├─Downsample: 1-8                                  [1, 192, 10, 10]         --
│    └─Conv2d: 2-28                                [1, 192, 10, 10]         590,016
├─SelfAttention2d: 1-9                             [1, 192, 10, 10]         --
│    └─GroupNorm: 2-29                             [1, 192, 10, 10]         384
│    └─MultiheadAttention: 2-30                    [1, 100, 192]            148,224
├─FiLMResBlock: 1-10                               [1, 384, 10, 10]         --
│    └─GroupNorm: 2-31                             [1, 192, 10, 10]         384
│    └─SiLU: 2-32                                  [1, 192, 10, 10]         --
│    └─Conv2d: 2-33                                [1, 384, 10, 10]         663,936
│    └─Sequential: 2-34                            [1, 768]                 --
│    │    └─SiLU: 3-5                              [1, 128]                 --
│    │    └─Linear: 3-6                            [1, 768]                 99,072
│    └─GroupNorm: 2-35                             [1, 384, 10, 10]         768
│    └─SiLU: 2-36                                  [1, 384, 10, 10]         --
│    └─Dropout2d: 2-37                             [1, 384, 10, 10]         --
│    └─Conv2d: 2-38                                [1, 384, 10, 10]         1,327,488
│    └─Conv2d: 2-39                                [1, 384, 10, 10]         74,112
├─Downsample: 1-11                                 [1, 384, 5, 5]           --
│    └─Conv2d: 2-40                                [1, 384, 5, 5]           2,359,680
```

```
├─FiLMResBlock: 1-12                          [1, 768, 5, 5]           --
│    └─GroupNorm: 2-41                        [1, 384, 5, 5]           768
│    └─SiLU: 2-42                             [1, 384, 5, 5]           --
│    └─Conv2d: 2-43                           [1, 768, 5, 5]           2,654,976
│    └─Sequential: 2-44                       [1, 1536]                --
│    │    └─SiLU: 3-7                         [1, 128]                 --
│    │    └─Linear: 3-8                       [1, 1536]                198,144
│    └─GroupNorm: 2-45                        [1, 768, 5, 5]           1,536
│    └─SiLU: 2-46                             [1, 768, 5, 5]           --
│    └─Dropout2d: 2-47                        [1, 768, 5, 5]           --
│    └─Conv2d: 2-48                           [1, 768, 5, 5]           5,309,184
│    └─Conv2d: 2-49                           [1, 768, 5, 5]           295,680
├─FiLMResBlock: 1-13                          [1, 768, 5, 5]           --
│    └─GroupNorm: 2-50                        [1, 768, 5, 5]           1,536
│    └─SiLU: 2-51                             [1, 768, 5, 5]           --
│    └─Conv2d: 2-52                           [1, 768, 5, 5]           5,309,184
│    └─Sequential: 2-53                       [1, 1536]                --
│    │    └─SiLU: 3-9                         [1, 128]                 --
│    │    └─Linear: 3-10                      [1, 1536]                198,144
│    └─GroupNorm: 2-54                        [1, 768, 5, 5]           1,536
│    └─SiLU: 2-55                             [1, 768, 5, 5]           --
│    └─Dropout2d: 2-56                        [1, 768, 5, 5]           --
│    └─Conv2d: 2-57                           [1, 768, 5, 5]           5,309,184
│    └─Identity: 2-58                         [1, 768, 5, 5]           --
├─FiLMResBlock: 1-14                          [1, 768, 5, 5]           --
│    └─GroupNorm: 2-59                        [1, 768, 5, 5]           1,536
│    └─SiLU: 2-60                             [1, 768, 5, 5]           --
│    └─Conv2d: 2-61                           [1, 768, 5, 5]           5,309,184
│    └─Sequential: 2-62                       [1, 1536]                --
│    │    └─SiLU: 3-11                        [1, 128]                 --
│    │    └─Linear: 3-12                      [1, 1536]                198,144
│    └─GroupNorm: 2-63                        [1, 768, 5, 5]           1,536
│    └─SiLU: 2-64                             [1, 768, 5, 5]           --
│    └─Dropout2d: 2-65                        [1, 768, 5, 5]           --
│    └─Conv2d: 2-66                           [1, 768, 5, 5]           5,309,184
│    └─Identity: 2-67                         [1, 768, 5, 5]           --
```

```
├─Upsample: 1-15                                  [1, 768, 10, 10]          --
│    └─ConvTranspose2d: 2-68                      [1, 768, 10, 10]          9,437,952
├─FiLMResBlock: 1-16                              [1, 384, 10, 10]          --
│    └─GroupNorm: 2-69                            [1, 1152, 10, 10]         2,304
│    └─SiLU: 2-70                                 [1, 1152, 10, 10]         --
│    └─Conv2d: 2-71                               [1, 384, 10, 10]          3,981,696
│    └─Sequential: 2-72                           [1, 768]                  --
│    │    └─SiLU: 3-13                            [1, 128]                  --
│    │    └─Linear: 3-14                          [1, 768]                  99,072
│    └─GroupNorm: 2-73                            [1, 384, 10, 10]          768
│    └─SiLU: 2-74                                 [1, 384, 10, 10]          --
│    └─Dropout2d: 2-75                            [1, 384, 10, 10]          --
│    └─Conv2d: 2-76                               [1, 384, 10, 10]          1,327,488
│    └─Conv2d: 2-77                               [1, 384, 10, 10]          442,752
├─Upsample: 1-17                                  [1, 384, 20, 20]          --
│    └─ConvTranspose2d: 2-78                      [1, 384, 20, 20]          2,359,680
├─FiLMResBlock: 1-18                              [1, 192, 20, 20]          --
│    └─GroupNorm: 2-79                            [1, 576, 20, 20]          1,152
│    └─SiLU: 2-80                                 [1, 576, 20, 20]          --
│    └─Conv2d: 2-81                               [1, 192, 20, 20]          995,520
│    └─Sequential: 2-82                           [1, 384]                  --
│    │    └─SiLU: 3-15                            [1, 128]                  --
│    │    └─Linear: 3-16                          [1, 384]                  49,536
│    └─GroupNorm: 2-83                            [1, 192, 20, 20]          384
│    └─SiLU: 2-84                                 [1, 192, 20, 20]          --
│    └─Dropout2d: 2-85                            [1, 192, 20, 20]          --
│    └─Conv2d: 2-86                               [1, 192, 20, 20]          331,968
│    └─Conv2d: 2-87                               [1, 192, 20, 20]          110,784
├─Upsample: 1-19                                  [1, 192, 40, 40]          --
│    └─ConvTranspose2d: 2-88                      [1, 192, 40, 40]          590,016
├─FiLMResBlock: 1-20                              [1, 96, 40, 40]           --
│    └─GroupNorm: 2-89                            [1, 288, 40, 40]          576
│    └─SiLU: 2-90                                 [1, 288, 40, 40]          --
│    └─Conv2d: 2-91                               [1, 96, 40, 40]           248,928
│    └─Sequential: 2-92                           [1, 192]                  --
│    │    └─SiLU: 3-17                            [1, 128]                  --
```

```
│     │     └─Linear: 3-18                              [1, 192]                  24,768
│     └─GroupNorm: 2-93                                 [1, 96, 40, 40]           192
│     └─SiLU: 2-94                                      [1, 96, 40, 40]           --
│     └─Dropout2d: 2-95                                 [1, 96, 40, 40]           --
│     └─Conv2d: 2-96                                    [1, 96, 40, 40]           83,040
│     └─Conv2d: 2-97                                    [1, 96, 40, 40]           27,744
├─GroupNorm: 1-21                                       [1, 96, 40, 40]           192
├─Conv2d: 1-22                                          [1, 1, 40, 40]            97
======================================================================================
Total params: 56,469,913
Trainable params: 56,469,913
Non-trainable params: 0
Total mult-adds (Units.GIGABYTES): 6.16
======================================================================================
```

6. Gradual generation of electric fields from the gaussian noise

This section presents examples about how electric field is gradually generated by the diffusion model. We will show 40 steps of the generation process. Actually, it is generated in 1000 steps. We use 40 equally divided steps for illustration purposes.

Firstly, we show an example from the design problem 1.

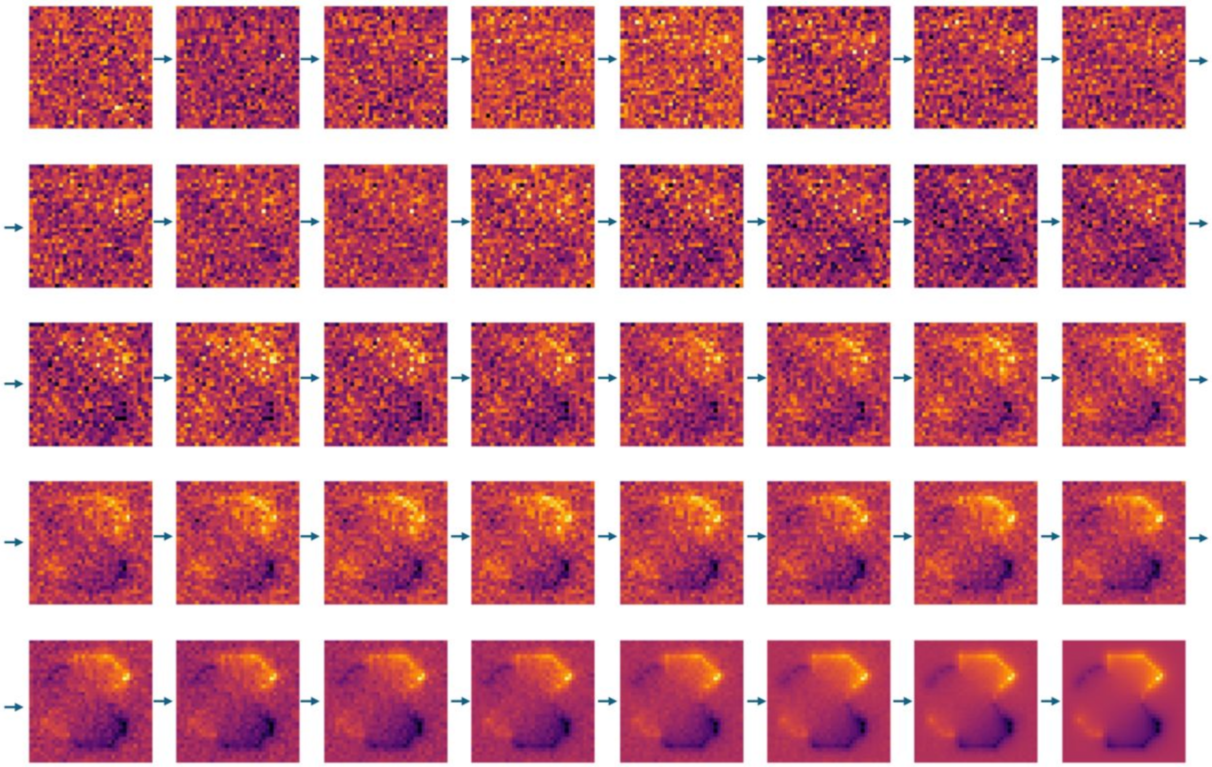

Figure S3: Electric field generation process as an intermediate output by the MxDiffusion framework for design problem 1. 40 steps are shown here. The top-left one is the true gaussian noise. Then from left to right, the noise starts to decrease. From the rightmost image, the next image is the leftmost image in the next line (row).

Now we show an example from design problem 2.

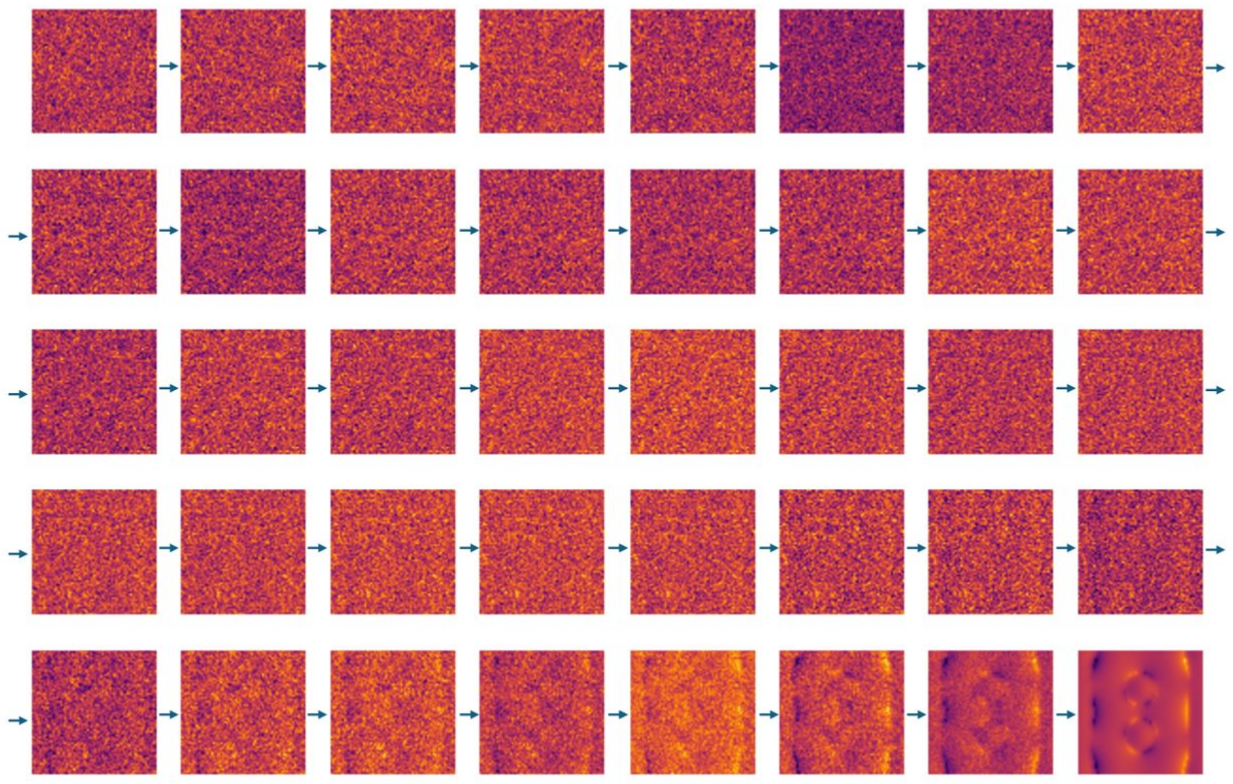

Figure S4: Electric field generation process as an intermediate output by the MxDiffusion framework for design problem 2. It is obvious that only the images in the final row contain some visible improvements in the generation process.

7. Gradual generation of final pattern from the gaussian noise

This section illustrates the final pattern generation by the diffusion model. Like section 5, 40 samples have been displayed.

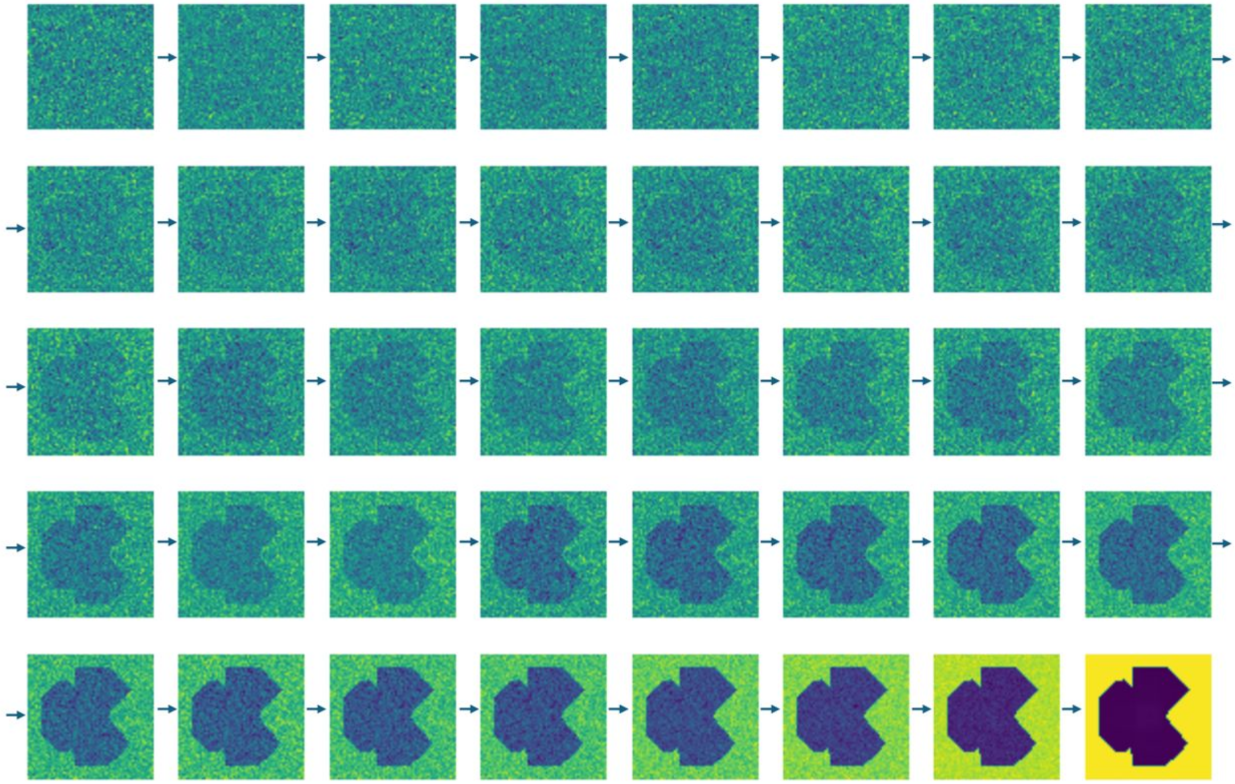

Figure S5: Final structure generation example for design problem 1. Target spectra and the electric field are used as the conditional inputs here. The generated patten is similar to the generated electric field in S3.

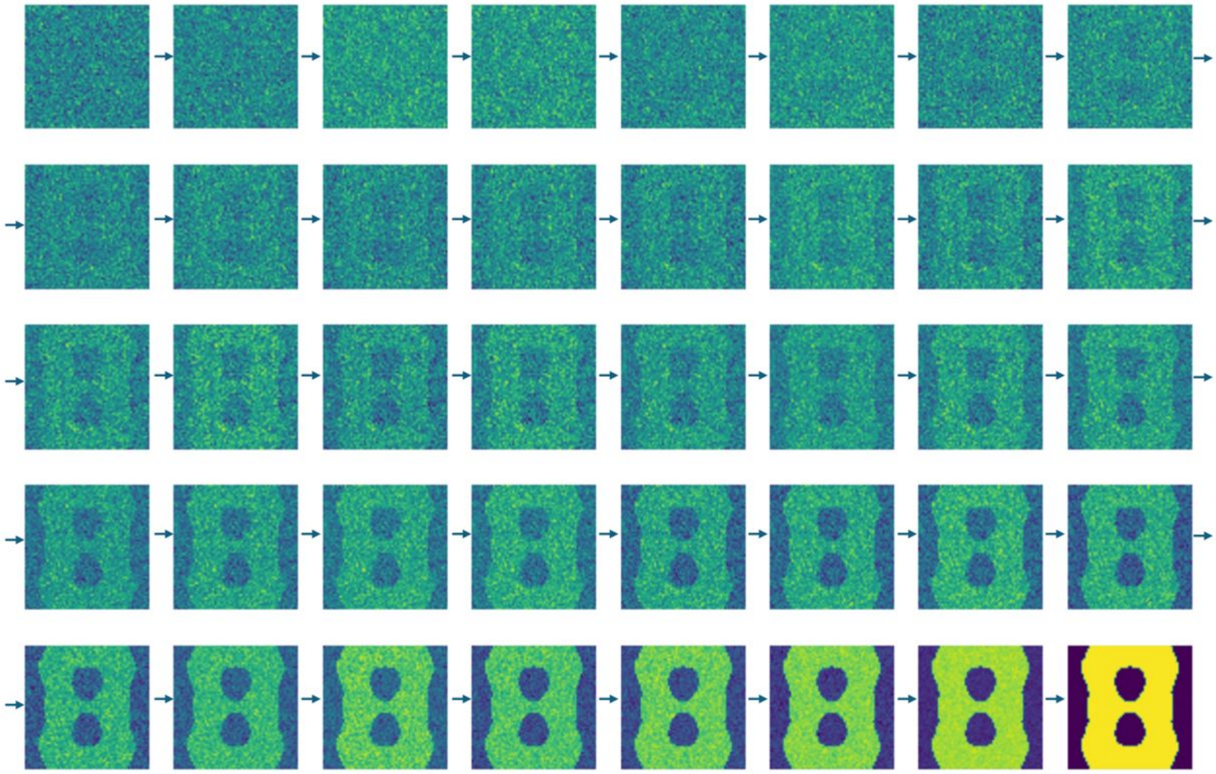

Figure S6: Final structure generation example for design problem 2. Target spectra and the electric field are used as the conditional inputs.

8. Timesteps selection and training procedure

To understand the timestep selection procedure for incorporating the Maxwell's Loss the readers are referred to Fig. S3 and Fig. S4, which illustrate that the electric field is progressively generated from random noise during the reverse diffusion process. The improved accuracy of our framework arises primarily from enhanced optimization of boundary regions under physical constraints. At earlier timesteps, the model focuses on capturing the global structure, whereas at later timesteps, finer boundary refinements are introduced. Maxwell's loss is particularly effective at smaller timesteps, where it enforces physical consistency and improves alignment with the target design objectives. The selection of timesteps for applying Maxwell's loss was determined empirically. We evaluated multiple intervals, including $t \in [0,200]$, [0,300], [0,500], and the full range [0,1000]. Based on extensive experimentation, we found that sampling approximately 75% of timesteps from the range [0,20] and 25% from [21,100] yields the most optimal performance, and results for this configuration are reported. We also observe that the model produces reliable results for other small-timestep ranges (e.g. [0,200],

[0,300]), indicating that the performance is not highly sensitive to the exact choice of timestep interval, provided that smaller timesteps are emphasized.

Our MxDiffusion framework uses two diffusion models, each of the models has almost same complexity. Our model was trained on NVIDIA GP104GL (Quadro P5000) GPU. Each of the models took less than 4 hours for the initial training. So, extra 4 hours of training was required for our framework compared to the normal diffusion model-based techniques. However, this training time requirement is a one-time thing and very trivial compared to the improvement achieved with this framework. After training, the sampling takes around 0.5 seconds for each target spectra for the normal diffusion model-based technique. While for our framework, it takes around one second. This is still trivial compared to the improvement achieved. Dataset generation does not introduce additional overhead compared to the data-driven approach; it only requires adding a DFT monitor to record the electric field. Since full-wave simulations inherently compute the field quantities, this addition does not increase simulation time. For storing the electric field, the full electric field dataset takes around 100MB of disk size, which is very minimal compared to the modern computation and storage capabilities.

References:

(1) Ho, J.; Jain, A.; Abbeel, P. Denoising diffusion probabilistic models. *Advances in Neural Information Processing Systems* **2020**, *33*, 6840-6851.
